# Challenges in healthcare financing for surgery in sub-Saharan Africa

**DOI:** 10.11604/pamj.2021.38.198.27115

**Published:** 2021-02-22

**Authors:** Juliet Siena Okoroh, Robert Riviello

**Affiliations:** 1 University of Alabama, Department of Surgery, Birmingham, Alabama, United States of America,; 2 Brigham and Women´s Hospital, Division of Trauma, Burn, Surgical Critical Care, Boston, Massachusetts, United States of America

**Keywords:** Health expenditures, healthcare financing, global health, sub-Saharan Africa, healthcare economics, healthcare organization, catastrophic illness

## Abstract

One-third of the global burden of disease is attributed to surgical conditions yet, 5 billion people globally, lack access to surgery. The Lancet Commission on Global Surgery, Obstetrics, and Anesthesia (LCOGS) published guidelines for improving access by reducing catastrophic health expenditures (CHEs) by 2030. This is especially important in sub-Saharan Africa (SSA) where 90% of the extreme poor reside. In this paper, we provide a narrative review of four studies on CHEs for surgical care in SSA published since 2015. We discuss healthcare financing in the countries and summarize the authors’ key findings of out-of-pocket payments (OOP) and CHEs. Briefly, the studies enrolled 130 to 300 patients and collected direct OOPs via chart review of health costs or patient interviews. Indirect costs were calculated from lost wages and transportation costs. CHEs were defined as health costs exceeding 10% of the GDP per capita or the household income. Despite healthcare being reported as free in all studies, 60%-90% of surgical patients had CHEs with all costs considered. OOPs persists for medicines and anesthesia that should be covered under any health insurance scheme. In some cases, indirect costs associated with transportation and wages were major drivers of CHEs for surgery. Without addressing these gaps in coverage, more people will risk impoverishment in seeking surgical care in SSA.

## Perspectives

It is estimated that one-third of the global burden of disease is attributed to surgical conditions, yet 5 billion people world-wide lack access to surgical care [1]. In 2015, The Lancet Commission on Global Surgery, Obstetrics, and Anesthesia (LCOGS) published a set of guidelines for achieving equitable surgical delivery within the context of Universal Health Coverage (UHC) which are centered on the following themes: access to timely and safe surgery, increasing surgical workforce density, increasing surgical volume in low- and middle-income countries (LMICs), reducing perioperative mortality rate, and protecting against impoverishing and catastrophic health expenditures (CHE) [2]. These guidelines have been incorporated into the World Bank Health Service Indicators in an effort to monitor progress in achieving these targets in all countries by 2030 [3]. Globally, 48 million people face financial catastrophe due to surgical care as a result of out-of-pocket payments (OOPs) [4]. This level of out-of-pocket spending has significant implications for the global agenda to end household poverty particularly in sub-Saharan Africa (SSA), where 90% of people living in extreme poverty reside [5]. Several countries in SSA including Nigeria, Ethiopia, Tanzania, and Rwanda have adopted national surgical, obstetric, and anesthesia plans (NSOAPs) to address the LCOGS´ targets; though these plans are at various stages of maturation. We recently published a paper evaluating the impact of the national health insurance scheme of Ghana (NHIS) on the risk of financial catastrophe for surgical care at a teaching hospital. We found that despite the benefits of NHIS, more than 60% of insured patients still risk facing financial catastrophe as a result of OOPs for surgical care [6]. We began to explore the experiences of countries in SSA with national health insurance schemes that include surgical care to develop recommendations for health reform in similar countries in the region. Since our focus is on health systems financing, the following countries were summarized given already published data on financial catastrophe and surgical care.

### Rwanda

Rwanda, a country of 12 million people in East Africa started a community-based health insurance scheme (CBHI) in 1999, five years after the end of the genocide in an attempt to rebuild the country´s healthcare infrastructure [7]. The program expanded from its pilot to covering close to 96% of the entire population as of 2012 [7]. The scheme is funded by household premiums (66%), the government (14%), the Global Fund to Fight AIDS, Tuberculosis and Malaria (10%), as well as co-payments (10%) which are assessed based on an individual´s ability to pay [8]. Premiums’ exemptions exist for the poor, elderly, and pregnant women. The plan covers a wide range of services including preventive medicine, maternal health, emergency, and surgical care. It has been recognized for the improvements catalyzed in antenatal care and the decrease in maternal mortality post-implementation [9,10]. Surgical care is based on a tiered system i.e., patients initially present to a primary facility and are subsequently referred to a district hospital or tertiary hospital where most surgical services are provided [11]. In 2017, Rickard *et al*. examined the impact of the health insurance scheme of Rwanda on the risk of CHEs on a cohort of 245 patients requiring surgical management of peritonitis over a six-month period at a tertiary hospital in Rwanda [12]. They calculated the total in-hospital charges for surgery and defined CHEs as healthcare payments exceeding 10% of Rwanda´s GDP per capita, which in 2017 was 700 USD. The median in-hospital charge was 308 USD however, as a result of insurance patients paid on average 27 USD out-of-pocket. This is 3.7% of the per capita GDP of Rwanda and not considered catastrophic. However, 13% of patients had medical costs which exceeded 10% of Rwanda´s GDP per capita. Similar to other studies in SSA, the largest in-patient cost categories were for medications (33%), including anesthesia, followed by procedural cost (33%), consumables (15%), and laboratory test (6%) [6,13,14]. Furthermore, including non-medical costs such as transportation costs and lost wages increased the proportion of individuals risking financial catastrophe up to 77% from 13%. The authors concluded that without CBHI, in-hospital charges alone for peritonitis would be catastrophic for most families in Rwanda. Non-medical costs, as well as OOPs for medicines not available in the public system remain a significant barrier to financial risk protection for many families.

### Ghana

Ghana, a middle-income country of 26 million people in West Africa has had over 14 years of experience with a national health insurance scheme (NHIS) that includes coverage of surgical care [15,16]. As of 2015, 38% of the Ghanaian population was actively enrolled with a particular interest by the government to cover vulnerable populations [17]. NHIS is a government-sponsored mandatory insurance plan funded by the National Health Insurance Levy which is a value-added tax on all goods and services in the country. This comprises 75% of the funding [18]. The rest comes from premium, registration, and social security deductions [18]. NHIS covers 95% of health conditions affecting Ghanaians, including a variety of in-patient and out-patient services. This includes surgeries, emergencies, and obstetric care [18]. Premiums are assessed based on ability to pay and exemptions exist for individuals over 70 years of age, pregnant women, children under 18, as well as pensioners. Currently, there are no co-payments at the point of care. As of 2015, more than 60% of the enrolled population were exempt from making payments making the NHIS one of the most equitable health insurance schemes in SSA [18].

Analyses of the NHIS over the last 14 years have found significant increase in the utilization of outpatient benefits and reduction in maternal mortality, yet several studies suggest that insured patients still risk financial catastrophe due to OOPs [19]. We published a study evaluating the impact of NHIS on the cost of surgical care in Ghana at a tertiary referral hospital [6]. The study enrolled 196 patients, collected in-hospital charges, and interviewed patients to identify gaps in coverage that were not included in the patients´ record. Financial catastrophe was defined as OOPs which exceeded 10% of annual household expenditures, 20% of the individual's income, or 40% of non-food expenditures. The average inpatient charge for surgical care was 626 USD. The insured paid on average 497 USD compared to 863 USD paid by the uninsured. The average annual income was 2000 USD. We found more than 60% of insured patients would risk financial catastrophe, spending on average 40% of their annual income on surgical care compared to 90% of the uninsured. Furthermore, anesthesia fees, medicines, and supplies posed specific challenges as they are not uniformly covered under the health insurance scheme [6,19].

### Malawi

Malawi, a low-income country of 17 million with 90% of individuals living in rural areas is one of the least developed countries in SSA, ranking 170 out of 174 countries in terms of life span [20]. More than 60% of the population live under 2 USD per day [21]. The government began healthcare reform via a Health Sector Wide Approach (Swap) since 2004 to deliver an essential health package which is inclusive of 11 diseases and conditions [22]. Healthcare financing is primarily through external funding (75%) with some funds from the Ministry of Health and OOPs [23,24]. Healthcare is delivered through a tiered network of government hospitals and faith-based hospitals with more specialized surgical care delivered at the tertiary hospital. There are no co-payments as healthcare is free at the government hospitals [25].

Bijlmakers *et al*. in 2019, conducted a cross-sectional survey of 137 patients across both district and tertiary hospitals who had a hernia operation in order to estimate the OOPs for hernia surgery and the risk of financial catastrophe [26]. CHEs were defined as OOPs for surgical care which exceeded 10% of the monthly income per capita. The per capita monthly income in the study ranged from less than 1 USD in the lowest quintile of social-economic status (SES) to 40 USD in the highest quintile. The total cost of surgery ranged from 30 USD to 90 USD with more than 80% of the costs as indirect costs such as lost wages and non-medical costs such as transportation costs. The authors found that 94% of patients at the district hospital and 87% of patients at the tertiary hospital would face financial catastrophe due to these costs. Furthermore, the authors examined the risk of financial catastrophe by quartiles of SES which demonstrated significant disparities. OOPs for patients in the lowest quartile was four times their per capita income (400%) while patients in the highest quartile spent only 13% of their per capita income on surgery. In addition, more than 30% of patients reported needing to borrow money or liquidate their assets to afford their bills. The authors concluded that though government-owned hospitals in Malawi do not charge any fees for surgery, patients do incur substantial costs in accessing surgery, which in many cases exceed their monthly incomes, particularly for the lower income quintiles. In a post-interview conducted at eight- and tenth-week intervals, 30% and 40% patients reported ongoing financial and functional limitations respectively suggesting that the economic and physical impact of surgical care can last beyond the immediate post-operative period.

### Uganda

Uganda is a landlocked country in East-Central Africa and has a population of over 42 million people. It began healthcare reform as early as 1999 with the passage of The National Health Policy Plan [27]. As of 2001, user fees were abolished at the point of care at all levels of care [27]. Healthcare is delivered through a networked of public, private non-profit, and for-profit hospitals. Close to 60% of health facilities in the country are public facilities where services are supposed to be delivered free at the point of care. The public hospitals are divided into general hospitals, regional hospital, and national referral hospitals. The World Bank estimates that 40% of Ugandan healthcare expenditure is OOP, and 80% of the population is at risk for financial catastrophe. Based on the World Bank definition of ‘extreme poverty’ (spending less the $1.90 per person per day, corrected for 2011 PP), one third of Ugandans live in extreme poverty [28].

A study conducted in 2016 at a tertiary government hospital interviewed patients undergoing C-section at the time of discharge, over a three-week period [29]. There were 295 patients interviewed, 182 of which had undergone cesarean section. A questionnaire was conducted with the aim of determining the rates of CHEs after surgery, defined as spending 10% of total annual expenses. In this study, 14% of patients faced financial catastrophe due to direct medical costs, and a third due to total direct costs which included transportation costs and feeding expenses. This study demonstrated that more than half of the patients had to take a loan, and just over a fifth had to liquidate their assets to pay for medical expenses. The authors concluded that despite healthcare being considered free at the government hospitals, 60% of surgical patients are pushed into extreme poverty every year from seeking care at this hospital.

### Discussion

These early cross-sectional studies provide us with a snapshot of how surgical services are being delivered, and the economic burden of accessing surgical care, in four SSA countries with an existing health plan for surgical care. The studies report significant costs associated with non-medical costs such as transportation fees and indirect costs such as lost wages which are typically not covered in insurance schemes. These costs are particularly impactful where rural-urban disparities exist as in the case of Malawi or if accessing surgical care is centralized at tertiary facilities as in Rwanda, Ghana, and Uganda. In the case of Rwanda, addition of the indirect costs increased the proportion of individuals who would face financial catastrophe from 13% to 77%. Similarly, in Malawi close to 90% of patients faced financial catastrophe due to indirect costs and there were significant cost disparities between accessing surgical care at the district vs tertiary hospitals. In Uganda close to 60% of patients would be impoverished due to access to surgical care. Primary considerations included broken equipment, cost of supplies, and cost of imaging services in Uganda which are currently not covered by the government.

The costs of anesthesia fees, medicines, and supplies appears to be an evolving theme across the studies. A WHO study of 39 LMICs showed that the cost of medicines constituted up to 60% of total inpatient costs at public facilities [30]. In Rwanda, medicines on average accounted for 30% of total inpatient cost (12). In Ghana, 40% of the total cost paid by the insured for surgical care was for medicines [6]. The WHO essential medication list encompasses more than 580 medications which include anesthetics and analgesics that are supposed to be available at the point of care worldwide, yet significant gaps exist in their availability and affordability in LMICs [31]. A study of over 600 facilities in Ghana, Kenya, and Uganda found that over 30% of essential medicines were out of stock, often including common antibiotics which are key to the provision of surgical care [32]. In a study of 50 essential medicines for non-communicable diseases (NCDs) in Malawi, authors found the average pricing in addition to the overall low-income of the patients contributed to the unaffordability of certain medications [33]. Ghana´s essential medication list includes 522 medications that are to be available at the point of care [34]. However, our study demonstrated that patients still paid for medications despite NHIS coverage [6]. Anectodical evidence suggests that this phenomenon may be due to in part to pharmacies refusing to accept NHIS due to poor reimbursements and preferring patients to pay out-of-pocket [6]. Furthermore, anesthesia fees which are critical to surgical management, are not included in the NHIS, further driving up the cost of care.

### Recommendations

Our findings are limited by the few studies available on this topic, thus it is important to note that more studies are needed to understand the geographic and socio-economic disparities that exist in the provision of surgical care through the tier-system. Based on our experience, any plan for universal health coverage inclusive of surgical care needs to be imbedded in providing access to primary health care and strengthening health services and referral patterns. Perhaps only patients requiring more complex operations are referred to the tertiary hospitals and more simple operations can be performed at the district hospitals at lower costs.

Gaps in the availability and affordability of medicines and coverage of anesthesia fees need to be addressed through improving procurement practices and pricing. This could include adopting quality generic substitutions for essential medicines and reducing trade markups. Furthermore, addressing indirect costs for health care by providing transportation vouchers or assisting families with accommodation in the form of cash transfer may be important for reducing the financial burden of healthcare. Lastly, increasing overall investments in health and health services research to meet the economic and growing burden of NCDs such as cancer and essential surgery is critical and timely. The Lancet Commission estimates that up to 420 billion USD are needed to scale up interventions in surgical care to a minimum of 5000 procedures per 100,000 to avert lost disability and productivity [1]. The World Bank estimates that it will be impossible to achieve UHC when countries spend less than 5% of the GDP on health care. Yet, many countries in SSA still invest less than 2% of their GDP on health care [35-37]. In contrast high income countries spend on average 10% of their GDP on health and overall have a lower incidence (2%) of catastrophic health payments [37,38].

### Conclusion

There has been considerable progress in the adoption of NSOAPs across SSA with a focus on addressing the mission of the LCOGS to reduce disability due to surgical disease. Yet OOPs persist for medicines and supplies that should be routinely covered under any national health insurance scheme, particularly in SSA where a growing number of the extremely poor reside. Socio-economic disparities persist and without adequate financial risk protection for vulnerable populations, more people will be pushed further below the international poverty line when accessing surgical care. Political and civil rights have to align with the mission to improve access to surgical care by strengthening health systems through universal health coverage. These countries provide a longitudinal experience and can serve as the basis for healthcare reform in SSA. Governments need to scale up the provision of essential surgical services inclusive of anesthesia care to meet the targets of the LCOGS by 2030.

### Funding

The authors have not declared a specific grant for this research from any funding agencies in the public, commercial or not-for-profit sectors.

## References

[1] Meara JG, Leather AJ, Hagander L, Alkire BC, Alonso N, Ameh EA (2015). Global Surgery 2030: evidence and solutions for achieving health, welfare, and economic development. Lancet.

[2] Alkire BC, Raykar NP, Shrime MG, Weiser TG, Bicker SW, Rose JA (2015). Global access to surgical care: a modelling study. Lancet Glob Health.

[3] World Bank Indicators (Health).

[4] Shrime MG, Dare AJ, Alkire BC, O´Neill K, Meara JG (2015). Catastrophic expenditure to pay for surgery worldwide: a modelling study. Lancet Glob Health.

[5] World Bank People in extreme poverty, People in extreme poverty. PovcalNet and Poverty & Equity Data Portal.

[6] Okoroh J, Sarpong DO, Essoun S, Riviello R, Harris H, Weissman JS (2020). Does insurance protect individuals from catastrophic payments for surgical care? An analysis of Ghana's National Health Insurance Scheme at Korle-Bu teaching Hospital. BMC Health Serv Res.

[7] The Rockefeller Foundation The Development of Community-Based Health Insurance in Rwanda: Experiences and Lessons.

[8] Fenny AP, Yates R, Thompson R (2020). Social health insurance schemes in Africa leave out the poor. Int Health.

[9] Hong R, Ayad M, Ngabo F (2011). Being insured improves safe delivery practices in Rwanda. J Community Health.

[10] Farmer PE, Nutt CT, Wagner CM, Sekabaraga C, Nuthulaganti J, Weigel JL (2013). Reduced premature mortality in Rwanda: lessons from success. BMJ.

[11] Ministry of Health, Government of Rwanda Third Health Sector Strategic Plan: July 2012-June 2018.

[12] Rickard JL, Ngarambe C, Ndayizeye L, Smart B, Majyambere JP, Riviello R (2018). Risk of Catastrophic Health Expenditure in Rwandan Surgical Patients with Peritonitis. World J Surg.

[13] Odhiambo J, Ruhumuriza J, Nkurunziza T, Riviello R, Shrime M, Lin Y (2019). Health Facility Cost of Cesarean Delivery at a Rural District Hospital in Rwanda Using Time-Driven Activity-Based Costing. Matern Child Health J.

[14] Cornelissen D, Mwapasa G, Gajewski J, McCauley T, Borgstein E, Brugha R (2018). The Cost of Providing District-Level Surgery in Malawi. World J Surg.

[15] Government of Ghana (2003). National Health Insurance Policy Framework.

[16] Ministry of health (2004). National Health Insurance Regulations, LI 1809.

[17] Mensah SA (2015). Chief Executive-National Health Insurance Authority. Presentation at Health Partner´s Summit (MOH).

[18] World Bank Group (2017). Ghana National Insurance Scheme. Improving Financial Sustainability Based on Expenditure Review.

[19] Okoroh J, Essoun S, Seddoh A, Harris H, Weissman JS, Selby-Dsane L (2018). Evaluating the impact of the national health insurance scheme of Ghana on out of pocket expenditures: a systematic review. BMC Health Serv Res.

[20] World Bank Group (2018). Malawi Systematic Country Diagnostic: Breaking the cycle of low growth and poverty reduction.

[21] World Bank Group (2017). Republic of Malawi Poverty Assessment.

[22] Zere E, Walker O, Kirigia J, Zawairra F, Magombo F, Kataika E (2010). Health financing in Malawi: Evidence from National Health Accounts. BMC Int Health Hum Rights.

[23] World Bank Group Health Nutrition and Population Statistics.

[24] Borghi J, Munthali S, Million LB, Martinez-Alvarez M (2018). Health financing at district level in Malawi: an analysis of the distribution of funds at two points in time. Health Policy Plan.

[25] Ministry of Health Republic of Malawi.

[26] Bijlmakers L, Wientjes M, Mwapasa G, Cornelissen D, Borgstein E, Broekhuizen H (2019). Out-of-pocket payments and catastrophic household expenditure to access essential surgery in Malawi-A cross-sectional patient survey. Ann Med Surg.

[27] Ministry of Health Uganda Healthcare financing review.

[28] The World Bank Group (2016). World Bank Open Data.

[29] Anderson GA, Ilcisin L, Kayima P, Abesiga L, Benitez NP, Ngonzi J (2017). Out-of-pocket payment for surgery in Uganda: The rate of impoverishing and catastrophic expenditure at a government hospital. PLOS ONE.

[30] World Health Organization (2010). Health services utilization and out-of-pocket expenditure at public and private facilities in low-income countries.

[31] WHO Model List of Essential Medicines.

[32] Masters SH, Burstein R, DeCenso B, Moore K, Haakenstad A, Ikilezi G (2014). Pharmaceutical availability across levels of care: evidence from facility surveys in Ghana, Kenya, and Uganda. PLOS ONE.

[33] Khuluza F, Haefele-Abah C (2019). The availability, prices and affordability of essential medicines in Malawi: A cross-sectional study. PLOS ONE.

[34] National Health Insurance Scheme Ghana Essential Medicine List.

[35] Mcintyre D, Meheus F, Rottingen JA (2017). What level of domestic government health expenditure should we aspire to for universal health coverage. Health Econ Policy Law.

[36] Watkins DA, Jamison DT, Mills A, Atun R, Danforth K, Glassman A, Jamison DT Universal Health Coverage and Essential Packages of Care (2017). Disease Control Priorities: Improving Health and Reducing Poverty.

[37] World Bank Data Repository.

[38] Wagstaff A, Flores G, Hsu J, Smith M, Chepynoga K, Buisman L (2018). Progress on catastrophic health spending in 133 countries: a retrospective observational study. Lancet Glob Health.

